# The Effect of Comorbidities and Complications on COVID-19 Mortality: A Detailed Retrospective Study in Western Romania

**DOI:** 10.3390/jpm13111552

**Published:** 2023-10-29

**Authors:** Monica Steluta Marc, Daniela Rosca, Felix Bratosin, Ovidiu Fira-Mladinescu, Cristian Oancea, Camelia Corina Pescaru, Diana Velescu, Norbert Wellmann, Alexandru Catalin Motofelea, Ioana Mihaiela Ciuca, Karina Saracin, Diana Manolescu

**Affiliations:** 1 Center for Research and Innovation in Precision Medicine of Respiratory Diseases, “Victor Babes” University of Medicine and Pharmacy Timisoara, Eftimie Murgu Square 2, 300041 Timisoara, Romania; marc.monica@umft.ro (M.S.M.); mladinescu@umft.ro (O.F.-M.); oancea@umft.ro (C.O.); pescaru.camelia@umft.ro (C.C.P.); velescu.diana@umft.ro (D.V.); dmanolescu@umft.ro (D.M.); 2 Department of Infectious Diseases, Discipline of Pulmonology, “Victor Babes” University of Medicine and Pharmacy Timisoara, Eftimie Murgu Square 2, 300041 Timisoara, Romania; 3 Doctoral School, “Victor Babes” University of Medicine and Pharmacy Timisoara, Eftimie Murgu Square 2, 300041 Timisoara, Romania; felix.bratosin@umft.ro (F.B.); norbert_wellmann@yahoo.com (N.W.); 4 Discipline of Infectious Diseases, “Victor Babes” University of Medicine and Pharmacy Timisoara, Eftimie Murgu Square 2, 300041 Timisoara, Romania; 5 Department of Internal Medicine, “Victor Babes” University of Medicine and Pharmacy Timisoara, Eftimie Murgu Square 2, 300041 Timisoara, Romania; alexandru.motofelea@umft.ro; 6 Department of Pediatrics, “Victor Babes” University of Medicine and Pharmacy Timisoara, Eftimie Murgu Square 2, 300041 Timisoara, Romania; ciuca.ioana@umft.ro; 7 Pediatric Pulmonology Unit, Clinical County Hospital, Evliya Celebi 1-3, 300226 Timisoara, Romania; 8 Emergency County Hospital Craiova, Strada Tabaci 1, 200642 Craiova, Romania; saracin.karina@yahoo.com; 9 Department of Radiology, “Victor Babes” University of Medicine and Pharmacy Timisoara, Eftimie Murgu Square 2, 300041 Timisoara, Romania

**Keywords:** COVID-19, SARS-CoV-2, survival analysis, comorbidity

## Abstract

COVID-19 continues to impact global health systems even after being declared over, with some patients exhibiting severe complications linked to pre-existing conditions. This study aimed to investigate the association between comorbidities, complications, and survival outcomes among COVID-19 survivors in Western Romania. Our hypothesis posited that comorbidities and complications significantly influence survival rates. We conducted a retrospective analysis of 1948 COVID-19 survivors admitted from January to December 2021, with 192 selected for detailed analysis based on inclusion and exclusion criteria. The severity of COVID-19 was classified according to WHO guidelines, and conditions like hypertension and obesity were defined using criteria from the European Society of Hypertension (ESH), the European Society of Cardiology (ESC), and WHO, respectively. Among the 192 patients, 33 had mild, 62 had moderate, and 97 had severe COVID-19. The median age across the severity groups was 63.2 years. Patients undergoing tracheostomy had a mortality rate of 83.3% versus 22.2% for non-tracheostomy patients (*p* < 0.001) and presented with significantly higher lung injury, hospitalization duration, and complications. Remarkably, tracheostomized patients were 17.50 times more likely to succumb to the disease (95% CI 4.39–116.91, *p* < 0.001). Furthermore, pneumothorax increased the mortality risk significantly (OR 22.11, 95% CI 5.72–146.03, *p* < 0.001). Intriguingly, certain conditions like grade I hypertension and grade II obesity showed a protective effect against mortality, whereas type 2 diabetes mellitus increased mortality risk (univariate OR 2.89, *p* = 0.001). The presence of certain comorbidities and complications significantly impacts the survival rates of COVID-19 patients in Western Romania. Notably, tracheostomy, pneumothorax, and T2DM were associated with increased mortality. This study underscores the importance of personalized patient care and provides insights for healthcare policymakers in Western Romania to improve clinical management strategies.

## 1. Introduction

Comorbidity, the presence of one or more additional conditions co-occurring with a primary condition, has been recognized as a significant factor influencing the severity and outcomes of COVID-19 [1,2,3,4,5,6,7,8,9]. Individuals with underlying health conditions such as hypertension, cardiovascular disorders, diabetes, and cerebrovascular diseases are reportedly at an elevated risk of severe disease manifestation, demanding escalated levels of medical intervention including, but not limited to, hospitalization, intensive care, and advanced respiratory support [9,10,11,12]. These comorbidities not only intensify the course of infection, but also contribute to heightened mortality rates, stressing the health care systems [11].

The ramifications of COVID-19 extend beyond the immediate consequences of the viral infection, impacting long-term physiological and psychological well-being. In the contemporary scenario, there is a pressing need to explore and elucidate the extensive spectrum of complications and comorbidities experienced by COVID-19 survivors, especially in diverse demographic settings, to tailor effective post-COVID care and management strategies [13]. Additionally, dissecting the intricate relationship between various comorbidities and COVID-19 outcomes can pave the way for enhanced clinical approaches and therapeutic protocols, potentially mitigating the disease’s impact.

Western Romania, being a part of a region with unique demographic and health characteristics, necessitates a focused study to understand the prevalence and impact of comorbidities and complications in COVID-19 survivors, considering the varied health care infrastructure, population density, and lifestyles. Tracheostomy, a commonly deployed intervention, is especially crucial to investigate given its frequency of utilization in severe cases of COVID-19 and its associated complications [14]. Delineating the complications associated with interventions like tracheostomy is instrumental in optimizing medical protocols and improving the overall quality of patient care [15,16].

This research is propelled by the need to explore the multifaceted dimensions of comorbidities and complications among COVID-19 survivors in Western Romania, thus providing localized insights that are critical for contextual and effective medical interventions. Evaluating the intricate interplay between comorbid conditions and COVID-19 is pivotal for predicting disease outcomes, tailoring treatment strategies, and allocating healthcare resources efficiently [17,18]. Enhanced knowledge in this domain is fundamental for augmenting clinical management strategies, public health policies, and patient care, thereby contributing to the broader fight against the COVID-19 pandemic.

Though various global guidelines have been instituted to address the myriad challenges posed by COVID-19, questions and dilemmas continue to surface, necessitating the continual refinement of medical approaches and procedures. The exigency to safeguard medical practitioners, especially those at elevated risk due to exposure to droplets, like ENT surgeons, emphasizes the need for rigorous procedural safety protocols, optimal personal protective equipment, and meticulous planning to balance resource allocation and patient needs [18].

This study hypothesizes that a significant correlation exists between the presence of comorbidities and the occurrence of complications, and that these factors considerably impact the survival rates among COVID-19 patients in Western Romania. The objectives of this study include the systematic evaluation of comorbidities and complications in COVID-19 survivors, the analysis of the effects of these factors on survival rates, and the identification of any associations between specific comorbidities, complications, and clinical outcomes in the target population. This investigation seeks to contribute to a more nuanced understanding of COVID-19 implications, aiming to inform and refine therapeutic interventions, clinical management, and healthcare policies in Western Romania.

## 2. Materials and Methods

### 2.1. Studied Patients

This research is a retrospective study, systematically evaluating consecutive COVID-19 survivors admitted from 1 January 2021 to 31 December 2021. This study received ethical approval from the Clinical Hospital of Infectious Diseases and Pulmonology “Victor Babes” Timisoara, and all participants provided written informed consent.

This investigation sourced comprehensive data from medical records, surgical reports, and hospital discharge summaries to appraise demographic details, comorbidities, and complications profiles. This exhaustive analysis aimed to uncover potential risk factors and examine their relationship with the survival rates of COVID-19 patients. The inclusion of substantial and diverse patient samples allowed for an inclusive and representative analysis, ensuring the reliability of the findings.

### 2.2. Study Measurements

**Figure 1:**
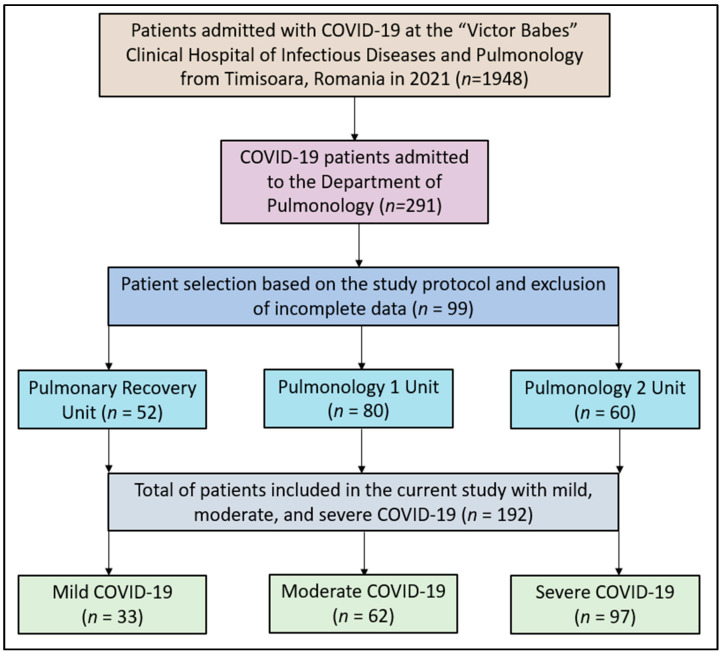
Study flowchart.

For this study, data were meticulously extracted from patient medical records, hospital databases, and interviews conducted with hospital staff regarding patient experiences with COVID-19. A total of 1948 confirmed COVID-19 cases from January 2021 to December 2021 were identified, from which 192 patients were selected for detailed analysis in the current study, as seen in Figure 1.

The classification of COVID-19 severity adhered to the criteria outlined by the World Health Organization (WHO), categorizing cases as mild, moderate, or severe based on symptoms, oxygen saturation levels, and imaging findings. Mild cases exhibited mild symptoms without pneumonia evidence; moderate cases displayed pneumonia symptoms without severe pneumonia signs; and severe cases involved respiratory failure, septic shock, and/or multiple organ dysfunction/failure [4,19].

Hypertension was categorized according to the guidelines from the European Society of Hypertension (ESH) and the European Society of Cardiology (ESC), with specific blood pressure ranges defining grade I and grade II hypertension [20]. Obesity classification relied on WHO’s BMI criteria, defining grade 1, 2, and 3 obesity based on specific body mass index (BMI) ranges [21].

The patients included in the study were 35 years or older, presenting with serious associated pathologies, or requiring disease-specific treatment for SARS-CoV-2 infections. This inclusion criterion was designed to examine the SARS-CoV-2 impact on populations vulnerable to severe symptoms and complications. Conversely, exclusion criteria encompassed patients below 35 years of age, those vaccinated against SARS-CoV-2, and emergency admissions, aiming to focus the study on unvaccinated individuals or those manifesting severe symptoms at admission.

By concentrating on patients with severe pathologies or those needing specific treatments for SARS-CoV-2 infections, this study sought to investigate the virus’s impact meticulously on populations more susceptible to severe manifestations and complications. The objective was to derive reliable and accurate associations between the severity, comorbidities, complications, and survival outcomes.

The independent variables in this study comprised the patient’s age, gender, comorbidities, the duration of hospitalization, and lung injury area. The dependent variable was considered the mortality after COVID-19, whereas the control variables used in the multivariate analysis included age, gender, comorbidities, area of lung injury, and tracheostomy placement.

### 2.3. Statistical Analysis

R software (version 3.6.3) was employed for statistical analysis. Continuous data were described as mean with standard deviation (SD) for normally distributed data and median with interquartile range for non-parametric data. Categorical data are represented as frequencies and percentages. For normally distributed continuous data, group differences were analyzed using Welch’s t-test or ANOVA depending on the number of groups. Non-parametric continuous data were assessed using the Mann–Whitney U test or Kruskal–Wallis test as appropriate, whereas categorical data differences were scrutinized using the χ^2^ test or Fisher’s exact test, contingent on expected cell counts. The Shapiro–Wilk test was employed to assess the normality of continuous data, verifying the underlying assumptions of normality for the statistical tests used.

To ascertain the influence of complications and comorbidities on COVID-19 mortality, univariate and multivariable analyses were conducted on odds ratio tables, adjusting for potential confounders. This involved the examination of the number and types of complications related to the dependent variable. Both univariate and multivariable models were constructed to isolate the independent effects of each variable on mortality, allowing for an in-depth analysis of the individual impact of each complication.

## 3. Results

At the end of the 2021 retrospective analysis of COVID-19 patients admitted in the Pulmonology Department, a total of 33 patients had mild disease, 62 were diagnosed with moderate SARS-CoV-2 infections, and the majority of 97 were considered severe. The age distribution among the categories showed that the median age was 60.4 years (IQR: 14.8) for patients with mild COVID-19, 65.8 years (IQR: 11.5) for those with moderate COVID-19, and 62.5 years (IQR: 11.5) for patients with severe symptoms. Overall, the median age of all the patients was 63.2 years (IQR: 12.2), but the differences in age distribution among the severity categories were not statistically significant (*p* = 0.085).

The median duration of hospital stay increased with the severity of the disease: 10.2 days (IQR: 5.0) for mild cases, 11.9 days (IQR: 8.3) for moderate cases, and 12.9 days (IQR: 9.6) for severe cases. However, the difference in hospital stay among the three categories was not statistically significant (*p* = 0.288). Males represented a substantial portion of patients across all severity levels, with 69.7% in the mild category, 61.3% in the moderate category, and 66.0% in the severe category. In total, 65.1% of the patients were males, and the gender distribution did not vary significantly across the severity categories (*p* = 0.692).

**Figure 2:**
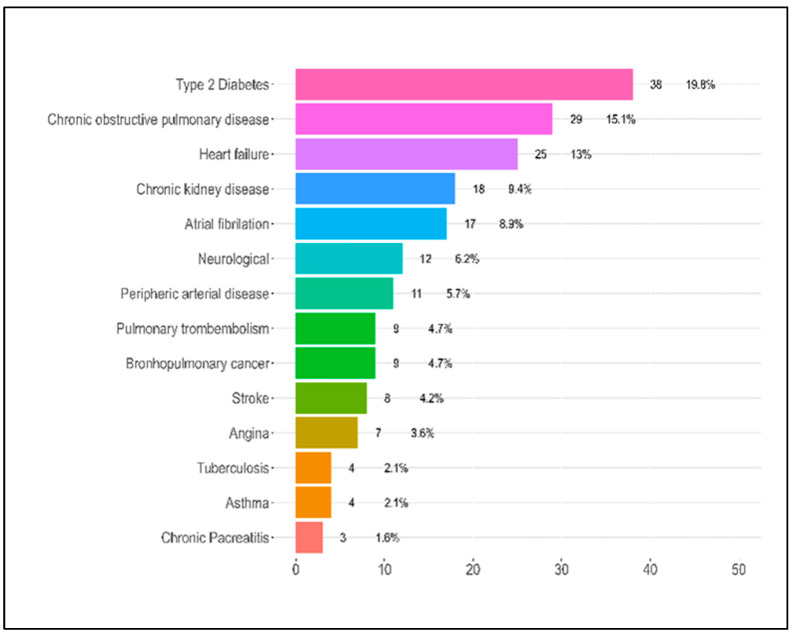
Patient comorbidities.

Several comorbidities were evaluated in the patients. Notably, the presence of coronary heart disease (CHD) had a statistically significant association with the severity of COVID-19, with 7.2% of severe cases having this condition compared to none in the mild and moderate groups (*p* = 0.029). The distribution of patients with chronic obstructive lung disease (COPD) also varied significantly across severity categories (*p* = 0.011). Interestingly, the severe category had a lower proportion of patients with COPD (8.2%) compared to the moderate category (25.8%), whereas the mild category had 15.2% of its patients with this condition. The distribution of patients who had suffered a stroke also varied significantly across the severity categories (*p* = 0.038). The highest percentage was seen in the mild category (12.1%), compared to 1.6% in the moderate category and 3.1% in the severe category. When considering the number of comorbidities per patient, there was a significant difference across the severity categories (*p* = 0.005). Specifically, a higher percentage of patients in the mild category (36.4%) had no comorbidities compared to the moderate (16.1%) and severe (8.2%) categories. Conversely, the severe category had the highest percentage of patients with 4–5 comorbidities (42.3%), as presented in Table 1 and Figure 2.

Table 2 offers insights into the complications experienced by patients during their hospitalization for COVID-19 based on the severity of their condition—mild, moderate, or severe. Regarding pulmonary complications, emphysema was seen in 30.3% of the patients with mild symptoms, 21% of those with moderate symptoms, and 16.5% of those with severe symptoms. The difference across these groups was not statistically significant (*p* = 0.232). Pulmonary thromboembolism was considerably higher in severe patients at 10.3% compared to moderate (3.2%) and mild cases (0.0%), with the *p*-value approaching significance (*p* = 0.052). Notably, cystic degeneration was exclusively found in severe patients at 8.2%, with a significant *p*-value of 0.017. Pulmonary superinfection had a unique distribution, with the highest rates observed in moderate cases (14.5%) and an unusually low percentage in severe cases (1.0%), resulting in a significant *p*-value of 0.003. Pneumothorax and pneumomediastinum were also significantly higher in the severe group, with *p*-values of 0.020 and 0.045, respectively.

For other complications, the presence of a pancreatic injury was statistically significant (*p* = 0.049), being more prevalent in the moderate group at 11.3%. Neurological complications and anxiety presented contrasting results, with the highest incidence in mild cases, 18.2% and 30.3%, respectively. Both complications displayed significant *p*-values, with 0.008 for neurological issues and 0.004 for anxiety. Tracheostomy and sepsis mirrored the trend observed for pulmonary thromboembolism, with higher incidences in severe cases and a *p*-value of 0.052 for each.

**Figure 3:**
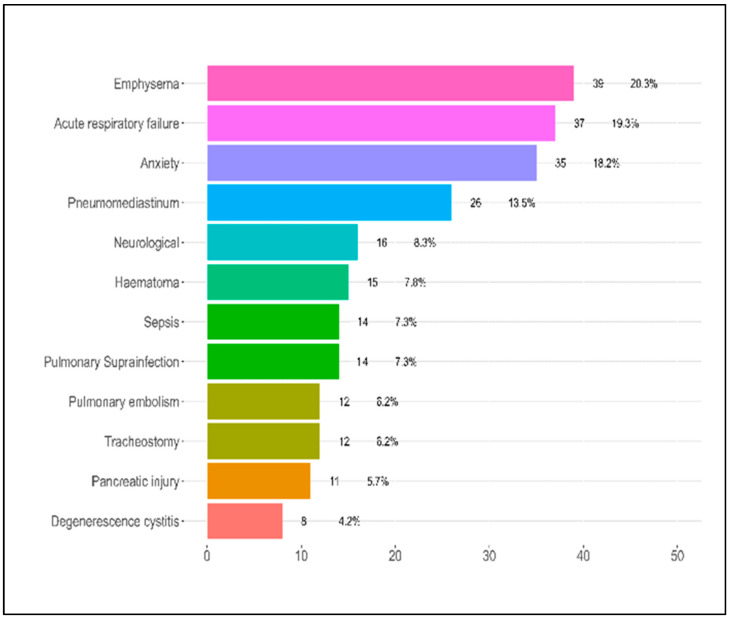
Patient complications during hospitalization.

Respiratory failure, although higher in severe cases (24.7%), did not reach a statistically significant difference across the groups, with a *p*-value of 0.108. However, the number of complications experienced by patients indicated a trend where patients with severe COVID-19 were more likely to have multiple complications, though the overall difference was not statistically significant (*p* = 0.155). Most alarmingly, the mortality rates exhibited a profound and statistically significant disparity across the groups. Patients with severe symptoms had a strikingly high mortality rate of 45.4%, in contrast to 8.1% in moderate and 3.0% in mild cases, as presented in Table 2 and Figure 3. The associated *p*-value was less than 0.001, highlighting the substantial differences in mortality rates based on the severity of COVID-19.

Table 3 presents the comparison of various parameters between patients who underwent tracheostomy and those who did not. Patients who underwent a tracheostomy had significantly longer hospitalization days, with a mean of 36.2 days (SD 11.3) compared to those without tracheostomy, who averaged 10.5 days (SD 5.4). This difference was statistically significant, with a *p*-value of <0.001. Age played a discernible role; those with a tracheostomy had a mean age of 52.3 years (SD 11.9), whereas the non-tracheostomy group averaged 63.9 years (SD 11.9), a significant age difference, with a *p*-value of 0.001.

Regarding lung injury, the area of lung injury, as seen on computed tomography (CT), was considerably higher in patients with a tracheostomy at 71.8% (SD 16.2) compared to those without at 55.8% (SD 17.5). This distinction was statistically significant, with a *p*-value of 0.002. Mortality rates were notably higher in the tracheostomy group, with 83.3% deceased compared to 22.2% in the non-tracheostomy group, presenting a highly significant *p*-value of <0.001. Respiratory failure was universally observed in tracheostomy patients (100%), whereas only 13.9% of patients without tracheostomy experienced it. This finding was statistically significant, with a *p*-value of <0.001.

Obesity presented intriguing disparities. Though the majority (74.4%) of patients without a tracheostomy had normal weight, a significant 41.7% of tracheostomized patients had obesity grade I. Overall, the distribution of obesity grades showed a significant difference between the groups, with a *p*-value of <0.001. For hypertension, the differences between the groups were not as pronounced, resulting in a non-significant *p*-value of 0.110. However, the number of complications displayed marked variations. Patients with tracheostomy showed a higher number of complications, with a staggering 58.3% having three complications compared to just 2.2% in the non-tracheostomy group. This was statistically significant, with a *p*-value of <0.001. Regarding the associated comorbidities, the tracheostomy group overwhelmingly had 4–5 comorbidities at 75.0%, in contrast to the non-tracheostomy group’s most common range of 1–3 comorbidities at 39.4%. The difference in the distribution of comorbidities was statistically significant, with a *p*-value of 0.003. Lastly, all tracheostomy patients (100%) had sepsis, a stark contrast to only 1.1% in the non-tracheostomy group. This difference was highly significant, with a *p*-value of <0.001.

Table 4 provides an assessment of the association between various comorbidities and COVID-19-related mortality. Starting with hypertension, patients with grade I hypertension displayed a noticeably lower mortality rate, at 9.1%, in comparison to those without it, at 39.4%. The univariate odds ratio (OR) for this group was 0.15 (95% CI 0.02–0.55, *p* = 0.013), suggesting a reduced risk. Even after adjusting for other variables in the multivariate analysis, the association remained significant, with an OR of 0.11 (95% CI 0.01–0.54, *p* = 0.015). For hypertension grade II, although the mortality rate was higher, at 43.1%, than those without it (30.5%), the difference was not statistically significant either in the univariate (OR 1.73, 95% CI 0.99–3.02, *p* = 0.053) or multivariate analysis (OR 1.25, 95% CI 0.56–2.79, *p* = 0.592). For grade III hypertension, similar non-significant associations were observed.

For obesity, grade I did not exhibit a significant association with mortality, but patients with grade II obesity showed a significant protective effect in the univariate analysis (OR 0.41, 95% CI 0.17–0.90, *p* = 0.035). This protection was further accentuated in the multivariate analysis (OR 0.29, 95% CI 0.11–0.73, *p* = 0.012). In terms of specific diseases, individuals with type 2 diabetes mellitus (T2DM) faced a notably higher risk of mortality (56.0% mortality rate) than those without (30.6%). The univariate OR was 2.89 (95% CI 1.52–5.56, *p* = 0.001) and remained significant in the multivariate analysis (OR 2.46, 95% CI 1.13–5.42, *p* = 0.024). Respiratory failure and chronic kidney disease (CKD) were also associated with a significantly increased risk of mortality, with multivariate ORs of 2.86 (95% CI 1.25–6.72, *p* = 0.014) and 7.54 (95% CI 1.84–36.68, *p* = 0.007), respectively. Heart failure was associated with an increased risk of mortality, significant in both univariate and multivariate analyses. For other comorbidities like lung cancer, atrial fibrillation, COPD (chronic obstructive lung disease), stroke, peripheral arterial disease, and tuberculosis, there was no statistically significant association with COVID-19 mortality in both univariate and multivariate analyses.

Table 5 examines the association between certain complications and COVID-19-related mortality. For patients who had a tracheostomy, mortality was significantly higher, with 83.3% succumbing to the disease compared to those without a tracheostomy, at 22.2%. The univariate odds ratio (OR) indicated that patients with a tracheostomy were 17.50 times more likely to die (95% CI 4.39–116.91, *p* < 0.001). When other factors were adjusted in a multivariate analysis, the odds were slightly lower, but still significant, with an OR of 9.89 (95% CI 1.78–81.48, *p* = 0.015).

Patients with respiratory insufficiency also showed an increased mortality rate of 51.4% compared to those without, at 20.0%. The univariate OR was 4.22 (95% CI 1.99–9.07, *p* < 0.001). However, after multivariate adjustments, the association became non-significant, with an OR of 2.10 (95% CI 0.73–5.69, *p* = 0.154). For pneumothorax, a stark difference was observed, with a mortality rate of 85.7% in those with the condition versus 21.3% in those without. The univariate OR was exceedingly high, at 22.11 (95% CI 5.72–146.03, *p* < 0.001), and this association remained robust even after multivariate adjustments, with an OR of 20.91 (95% CI 4.84–147.17, *p* < 0.001). Pneumomediastinum patients had a mortality rate of 38.5%, contrasted with 24.1% for those without the condition. The univariate OR was 1.97 (95% CI 0.81–4.64, *p* = 0.125). However, the multivariate analysis yielded an OR of 2.38 (95% CI 0.87–6.47, *p* = 0.088), suggesting a non-significant trend toward higher mortality.

Interestingly, emphysema appeared protective against COVID-19-related mortality. Only 10.3% of patients with emphysema died compared to 30.1% of those without. The univariate OR was 0.27 (95% CI 0.08–0.71, *p* = 0.017), indicating a significantly reduced risk. Yet, after multivariate adjustments, the association became non-significant, with an OR of 0.40 (95% CI 0.10–1.25, *p* = 0.141).

## 4. Discussion

### 4.1. Literature Findings

The present retrospective study provides a comprehensive insight into the clinical characteristics, comorbidities, complications, and mortality rates associated with COVID-19 among patients admitted to the Pulmonology Department in Western Romania in 2021. Most of the patients presented with severe COVID-19, with a median age of around the early 60s across all severity levels. Interestingly, though one might anticipate a trend in age differences correlating with disease severity, our data did not demonstrate a statistically significant difference among the severity categories. A noteworthy observation was the prominent male predominance across all disease severities, a finding that has been replicated in other studies, suggesting a potentially heightened vulnerability among males to SARS-CoV-2 infections [22,23].

Comorbidity analysis revealed significant associations between disease severity and certain underlying health conditions. Particularly, the presence of CHD was significantly associated with severe COVID-19. A stark contrast was seen in COPD, where a higher prevalence was observed in moderate cases compared to severe ones. The link between prior stroke and mild COVID-19 severity was an intriguing find, warranting further investigations into the potential mechanisms behind this association.

Complications during hospitalization varied considerably across disease severities. The exclusive presence of cystic degeneration in severe patients and the higher prevalence of pulmonary thromboembolism and pneumothorax align with the understanding that more severe infections can manifest with complex lung pathologies. Remarkably, pulmonary superinfections were most prevalent in moderate cases, which could be indicative of the unique interplay between viral-induced lung damage and secondary bacterial infections in these patients. Other complications, such as pancreatic injuries and neurological issues, revealed an unexpected trend towards higher prevalence in moderate and mild cases, respectively. The heightened incidence of anxiety in mild cases could be attributed to the psychological stress induced by the pandemic and associated uncertainties.

Multivariate analysis assessing the association between various complications and mortality provided compelling findings. Though conditions like pneumothorax exhibited strong associations with increased mortality, intriguingly, emphysema and anxiety seemed to offer some protective effects, even though the significance diminished after adjustments. The protective effect observed with anxiety is intriguing and may highlight the potential role of heightened health vigilance among these patients, though more research is required to substantiate this hypothesis. Comorbidity-related mortality assessment exhibited a protective effect for patients with grade I hypertension and grade II obesity, contrary to prevailing assumptions about these conditions exacerbating COVID-19 outcomes. The heightened mortality risk among T2DM patients resonates with the existing literature linking poor glycemic control with adverse COVID-19 outcomes [24,25].

Undoubtedly, one of the most concerning findings was the significant disparity in mortality rates based on disease severity. The mortality rate of 45.4% in severe cases underscores the critical nature of advanced SARS-CoV-2 infections and the imperative need for early interventions [26,27,28]. The analysis concerning patients undergoing tracheostomy revealed longer hospital stays, larger lung injury areas on CT scans, higher comorbidity rates, and notably higher mortality rates compared to non-tracheostomized patients. These observations underscore the critical condition of patients requiring tracheostomy and emphasize the need for aggressive monitoring and interventions for this subgroup.

Tracheostomy, a standard surgical intervention, aids secretion clearance, facilitates ventilation, and potentially reduces intensive care unit stays. This procedure is commonly executed either as an emergency response to upper airway obstruction or for patients requiring prolonged intubation [29]. A notable study by Abe et al. in 2018 revealed that tracheostomies were performed on 13% of ARDS cases, and in 75% of these cases, the surgery was conducted after the seventh day of the illness [30].

Complications linked to tracheostomies are well-documented. A study by Bontempo et al. cited a complication rate of 40–50%, distinguishing them into early or late complications [31]. These complications can range from hemorrhages and infections in the early stages [17,32] to tracheal stenosis and aspiration in the later stages [17,33]. In contrast, a study by Murray et al. on nearly 700 tracheotomized patients between 2011 and 2018 reported only a 10% complication rate [34].

The unprecedented demand for mechanical ventilation due to the surge in COVID-19 cases steered many otorhinolaryngological societies to devise new guidelines and policies [35,36]. In regions with limited ventilator availability, tracheostomies became a common practice. Our institution’s approach was multidisciplinary, ensuring tracheostomies were performed in adherence to national guidelines by senior ENT surgeons using protective measures.

Controversy surrounds the optimal timing of tracheostomies in COVID-19 patients. Some early anecdotal reports recommended performing the procedure after 21 days post-symptom onset [18,37,38,39]. However, a 2023 retrospective study on over 5000 COVID-19 patients intubated revealed a lower mortality rate for those who underwent a tracheostomy, specifically indicating that a tracheostomy on day 11 was linked to reduced mortality [40]. This finding contradicted other studies suggesting that earlier procedures increased mortality risks [41].

Regarding the procedure’s safety, a 2021 study from France found no infections among the surgical team post-tracheostomies, with patient postoperative complications standing at 15% [36]. Similarly, a study in 2022 by Hansson et al. reported postoperative complications in 36% of their COVID-19 cohort, highlighting bleeding as a prevalent issue [39,42,43]. Interestingly, a comprehensive 2022 meta-analysis, encompassing data from over 1000 studies, determined a cumulative complication incidence of 14.24%, with hemorrhages constituting 52% of all complications [44]. Despite the pandemic’s dynamic nature and evolving guidelines, the international consensus emphasized tracheostomy’s safety and its potential benefits for COVID-19 patients [45,46,47,48].

Our study indicates that in Eastern Europe, age, comorbidities, and complications significantly influence the survival rates of COVID-19 patients. Notably, younger patients with fewer comorbidities and complications demonstrated higher survival rates. The predominant complications observed include emphysema, respiratory failure, anxiety, and pneumomediastinum. The most frequent comorbidities were type 2 diabetes, COPD, and heart failure. Interestingly, our data showed a higher incidence of pancreatic injury in the moderate lung damage group, highlighting the potential role of overall health and immunity rather than the extent of lung damage. Moreover, recent studies confirm the exacerbation of mortality risk due to factors like advanced age, existing conditions, and certain socio-economic determinants [49,50,51,52,53,54,55,56,57,58,59,60,61].

A distinct finding in our research was the lower incidence of pneumomediastinum, at 0.3%, compared to the general COVID-19-related pneumothorax incidence of 7.3% noted in other studies [52,53]. Cardiovascular diseases surfaced as the primary contributors to increased mortality rates, and our observations concerning neurological complications and pulmonary infections were lower than in previous research [54,55,56,57,58]. Secondary pulmonary infections were mainly prevalent in critically ill COVID-19 patients, emphasizing the potential challenges in treating patients with these comorbidities [57,58].

Another pivotal aspect of our study involved analyzing various independent variables’ correlations with COVID-19 mortality rates. Utilizing both univariate and multivariate approaches, the initial analysis indicated respiratory failure and type 2 diabetes as major factors linked with increased mortality [62,63,64]. However, adjusting for other factors in multivariate analysis nuanced these associations. Whereas some relationships, like that between respiratory failure and mortality, became less evident, others, like the connection between type 2 diabetes and mortality, persisted [65,66,67]. Lastly, to optimize COVID-19 survival rates, we advocate for multifaceted approaches. Addressing socio-economic disparities, fostering health-conscious behaviors, ensuring access to timely medical care, and researching complications linked with increased mortality are crucial strategies. Further studies are vital to delineate the determinants of COVID-19 survival rates and pinpoint effective interventions [68,69,70].

### 4.2. Study Limitations

Several limitations were inherent in this study. Firstly, being a retrospective analysis, this study was subject to the confines of available medical records and data, possibly resulting in missing or incomplete information. Although this study spanned the entire year of 2021, it focused on a single healthcare center, potentially limiting the generalizability of the findings to wider populations or different healthcare settings. This study’s decision to exclude vaccinated individuals and those below 35 years of age precludes insights into the effects of vaccination on COVID-19 severity and the disease’s impact on younger populations. Furthermore, though the inclusion criteria of selecting patients 35 years or older with severe associated pathologies provides valuable insights into high-risk populations, it omits data on the broader spectrum of COVID-19 patients. Moreover, the results of the current study can be biased by having selected only hospitalized patients in the analysis, who might present with a higher disease severity and, therefore, more complications can be expected. Finally, despite the comprehensive statistical analyses, unmeasured confounding factors could still be present, influencing the observed associations.

## 5. Conclusions

In Western Romania, survival outcomes for COVID-19 patients are heavily influenced by the presence of specific comorbidities and complications. Our study found that undergoing a tracheostomy or encountering complications like pneumothorax significantly heightened the mortality risk among patients. On the other hand, certain conditions, notably grade I hypertension and grade II obesity, appeared to confer a protective effect against fatal outcomes, a finding that warrants further exploration. Conversely, the presence of type 2 diabetes mellitus was shown to increase the likelihood of mortality. These insights, which emphasize the intricacies of patient health profiles and their correlation with COVID-19 outcomes, underscore the pressing need for personalized patient care strategies.

## Figures and Tables

**Table 1:** Background characteristics of patients with mild, moderate, and severe COVID-19.

Variables	Mild (n = 33)	Moderate (n = 62)	Severe (n = 97)	Total (n = 192)	*p*-Value
Age (median, IQR)	60.4 (14.8)	65.8 (11.5)	62.5 (11.5)	63.2 (12.2)	0.085
Days of hospital stay (median, IQR)	10.2 (5.0)	11.9 (8.3)	12.9 (9.6)	12.1 (8.6)	0.288
Male gender n (%)	23 (69.7)	38 (61.3)	64 (66.0)	125 (65.1)	0.692
Comorbidities					
Cardiovascular n (%)					
Atrial fibrillation	2.0 (6.1%)	4.0 (6.5%)	11.0 (11.3%)	17.0 (8.9%)	0.471
CHD	0.0 (0.0%)	0.0 (0.0%)	7.0 (7.2%)	7.0 (3.6%)	0.029
Hypertension					0.071
Without hypertension	8.0 (24.2%)	18.0 (29.0%)	27.0 (27.8%)	53.0 (27.6%)	
Hypertension grade I	8.0 (24.2%)	3.0 (4.8%)	13.0 (13.4%)	24.0 (12.5%)	
Hypertension grade II	12.0 (36.4%)	35.0 (56.5%)	39.0 (40.2%)	86.0 (44.8%)	
Hypertension grade III	5.0 (15.2%)	6.0 (9.7%)	18.0 (18.6%)	29.0 (15.1%)	
Heart failure	4.0 (12.1%)	7.0 (11.3%)	14.0 (14.4%)	25.0 (13.0%)	0.836
Obesity n (%)					0.117
Normal weight	27.0 (81.8%)	44.0 (71.0%)	67.0 (69.1%)	138.0 (71.9%)	
Obesity grade I	0.0 (0.0%)	4.0 (6.5%)	10.0 (10.3%)	14.0 (7.3%)	
Obesity grade II	6.0 (18.2%)	14.0 (22.6%)	15.0 (15.5%)	35.0 (18.2%)	
Obesity grade III	0.0 (0.0%)	0.0 (0.0%)	5.0 (5.2%)	5.0 (2.6%)	
Peripheric arterial disease	0.0 (0.0%)	2.0 (3.2%)	9.0 (9.3%)	11.0 (5.7%)	0.083
Pulmonary n (%)					
Asthma	0.0 (0.0%)	1.0 (1.6%)	3.0 (3.1%)	4.0 (2.1%)	0.534
Lung Cancer	2.0 (6.1%)	3.0 (4.8%)	4.0 (4.1%)	9.0 (4.7%)	0.900
COPD	5.0 (15.2%)	16.0 (25.8%)	8.0 (8.2%)	29.0 (15.1%)	0.011
Tuberculosis	0.0 (0.0%)	0.0 (0.0%)	4.0 (4.1%)	4.0 (2.1%)	0.135
Thromboembolism	0.0 (0.0%)	2.0 (3.2%)	7.0 (7.2%)	9.0 (4.7%)	0.191
Others n (%)					
T2DM	6.0 (18.2%)	13.0 (21.0%)	19.0 (19.6%)	38.0 (19.8%)	0.946
CKD	2.0 (6.1%)	4.0 (6.5%)	12.0 (12.4%)	18.0 (9.4%)	0.354
Neurological	5.0 (15.2%)	2.0 (3.2%)	9.0 (9.3%)	16.0 (8.3%)	0.120
Stroke	4.0 (12.1%)	1.0 (1.6%)	3.0 (3.1%)	8.0 (4.2%)	0.038
Chronic pancreatitis	0 (0.0)	0 (0.0)	3 (3.1)	3 (1.6)	0.225
Number of comorbidities					0.005
No comorbidities	12.0 (36.4%)	10.0 (16.1%)	8.0 (8.2%)	30.0 (15.6%)	
1–3 comorbidities	6.0 (18.2%)	27.0 (43.5%)	38.0 (39.2%)	71.0 (37.0%)	
4–5 comorbidities	11.0 (33.3%)	18.0 (29.0%)	41.0 (42.3%)	70.0 (36.5%)	
6–8 comorbidities	4.0 (12.1%)	7.0 (11.3%)	10.0 (10.3%)	21.0 (10.9%)	

IQR—interquartile range; COPD—chronic obstructive lung disease; CHD—coronary heart disease; CKD—chronic kidney disease; T2DM—type 2 diabetes mellitus. Proportions were compared using the chi-squared test or Fisher’s exact test if the frequency assumption was not met.

**Table 2:** Complications during hospitalization for COVID-19.

Variables	Mild (n = 33)	Moderate (n = 62)	Severe (n = 97)	Total (n = 192)	*p*-Value
Pulmonary complications					
Emphysema	10 (30.3)	13 (21.0)	16 (16.5)	39 (20.3)	0.232
Pulmonary thromboembolism	0 (0.0)	2 (3.2)	10 (10.3)	12 (6.2)	0.052
Cystic degeneration	0 (0.0)	0 (0.0)	8 (8.2)	8 (4.2)	0.017
Pulmonary superinfection	4 (12.1)	9 (14.5)	1 (1.0)	14 (7.3)	0.003
Pneumothorax	0 (0.0)	2 (3.2)	12 (12.4)	14 (7.3)	0.020
Pneumomediastinum	2 (6.1)	5 (8.1)	19 (19.6)	26 (13.5)	0.045
Others					
Digestive hemorrhage	2 (6.1)	2 (3.2)	0 (0.0)	4 (2.1)	0.081
Pancreatic injury	0 (0.0)	7 (11.3)	4 (4.1)	11 (5.7)	0.049
Hematoma	0 (0.0)	7 (11.3)	8 (8.2)	15 (7.8)	0.145
Neurological complications	6 (18.2)	2 (3.2)	4 (4.1)	12 (6.2)	0.008
Anxiety	10 (30.3)	16 (25.8)	9 (9.3)	35 (18.2)	0.004
Tracheostomy	0 (0.0)	2 (3.2)	10 (10.3)	12 (6.2)	0.052
Sepsis	0 (0.0)	2 (3.2)	10 (10.3)	12 (6.2)	0.052
Respiratory failure	3.0 (9.1%)	10.0 (16.1%)	24.0 (24.7%)	37.0 (19.3%)	0.108
Number of complications					0.155
1	26.0 (78.8%)	43.0 (69.4%)	59.0 (60.8%)	128.0 (66.7%)	
2	7.0 (21.2%)	17.0 (27.4%)	29.0 (29.9%)	53.0 (27.6%)	
3	0.0 (0.0%)	2.0 (3.2%)	9.0 (9.3%)	11.0 (5.7%)	
Mortality	1 (3.0)	5 (8.1)	44 (45.4)	50 (26.0)	<0.001

Proportions were compared using the chi-squared test or Fisher’s exact test if the frequency assumption was not met.

**Table 3:** Comparison of parameters in patients with and without tracheostomy.

Dependent: Tracheostomy		No	Yes	Total	*p*-Value
Hospitalization days	Mean (SD)	10.5 (5.4)	36.2 (11.3)	12.1 (8.6)	<0.001
Age	Mean (SD)	63.9 (11.9)	52.3 (11.9)	63.2 (12.2)	0.001
Area of lung injury on CT	Mean (SD)	55.8 (17.5)	71.8 (16.2)	56.8 (17.8)	0.002
Deceased	No	140 (77.8)	2 (16.7)	142 (74.0)	<0.001
	Yes	40 (22.2)	10 (83.3)	50 (26.0)	
Respiratory failure	No	155 (86.1)	0 (0.0)	155 (80.7)	<0.001
	Yes	25 (13.9)	12 (100.0)	37 (19.3)	
Obesity	Normal weight	134 (74.4)	4 (33.3)	138 (71.9)	<0.001
	Obesity grade I	9 (5.0)	5 (41.7)	14 (7.3)	
	Obesity grade II	34 (18.9)	1 (8.3)	35 (18.2)	
	Obesity grade III	3 (1.7)	2 (16.7)	5 (2.6)	
Hypertension	Without hypertension	53 (29.4)	0 (0.0)	53 (27.6)	0.110
	Hypertension grade I	21 (11.7)	3 (25.0)	24 (12.5)	
	Hypertension grade II	80 (44.4)	6 (50.0)	86 (44.8)	
	Hypertension grade III	26 (14.4)	3 (25.0)	29 (15.1)	
Number of complications	1	128 (71.1)	0 (0.0)	128 (66.7)	<0.001
	2	48 (26.7)	5 (41.7)	53 (27.6)	
	3	4 (2.2)	7 (58.3)	11 (5.7)	
Number of comorbidities	No comorbidities	30 (16.7)	0 (0.0)	30 (15.6)	0.003
	1–3 comorbidities	71 (39.4)	0 (0.0)	71 (37.0)	
	4–5 comorbidities	61 (33.9)	9 (75.0)	70 (36.5)	
	6–8 comorbidities	18 (10.0)	3 (25.0)	21 (10.9)	
Sepsis	No	178 (98.9)	0 (0.0)	178 (92.7)	<0.001
	Yes	2 (1.1)	12 (100.0)	14 (7.3)	

CT—computed tomography. Proportions were compared using the chi-squared test or Fisher’s exact test if the frequency assumption was not met.

**Table 4:** Association between comorbidities and COVID-19 mortality.

Dependent: Mortality		Survived	Died	OR (Univariate)	OR (Multivariate)
Hypertension grade I	No	120 (60.6)	78 (39.4)	-	-
	Yes	20 (90.9)	2 (9.1)	0.15 (0.02–0.55, *p* = 0.013)	0.11 (0.01–0.54, *p* = 0.015)
Hypertension grade II	No	82 (69.5)	36 (30.5)	-	-
	Yes	58 (56.9)	44 (43.1)	1.73 (0.99–3.02, *p* = 0.053)	1.25 (0.56–2.79, *p* = 0.592)
Hypertension grade III	No	122 (65.6)	64 (34.4)	-	-
	Yes	18 (52.9)	16 (47.1)	1.69 (0.80–3.55, *p* = 0.161)	1.61 (0.59–4.37, *p* = 0.349)
Obesity grade I	No	132 (62.9)	78 (37.1)	-	-
	Yes	8 (80.0)	2 (20.0)	0.42 (0.06–1.74, *p* = 0.284)	0.18 (0.02–1.27, *p* = 0.121)
Obesity grade II	No	110 (60.4)	72 (39.6)	-	-
	Yes	30 (78.9)	8 (21.1)	0.41 (0.17–0.90, *p* = 0.035)	0.29 (0.11–0.73, *p* = 0.012)
Lung cancer	No	136 (64.2)	76 (35.8)	-	-
	Yes	4 (50.0)	4 (50.0)	1.79 (0.41–7.76, *p* = 0.420)	2.35 (0.38–15.17, *p* = 0.347)
Atrial fibrillation	No	128 (65.3)	68 (34.7)	-	-
	Yes	12 (50.0)	12 (50.0)	1.88 (0.80–4.46, *p* = 0.146)	1.35 (0.44–4.04, *p* = 0.593)
T2DM	No	118 (69.4)	52 (30.6)	-	-
	Yes	22 (44.0)	28 (56.0)	2.89 (1.52–5.56, *p* = 0.001)	2.46 (1.13–5.42, *p* = 0.024)
Respiratory failure	No	124 (67.4)	60 (32.6)	-	-
	Yes	16 (44.4)	20 (55.6)	2.58 (1.25–5.40, *p* = 0.010)	2.86 (1.25–6.72, *p* = 0.014)
CKD	No	132 (66.7)	66 (33.3)	-	-
	Yes	8 (36.4)	14 (63.6)	3.50 (1.43–9.16, *p* = 0.007)	7.54 (1.84–36.68, *p* = 0.007)
Heart failure	No	124 (66.7)	62 (33.3)	-	-
	Yes	16 (47.1)	18 (52.9)	2.25 (1.07–4.76, *p* = 0.032)	3.43 (1.36–9.15, *p* = 0.011)
COPD	No	120 (65.2)	64 (34.8)	-	-
	Yes	20 (55.6)	16 (44.4)	1.50 (0.72–3.09, *p* = 0.272)	1.85 (0.73–4.71, *p* = 0.191)
Stroke	No	134 (63.8)	76 (36.2)	-	-
	Yes	6 (60.0)	4 (40.0)	1.18 (0.29–4.24, *p* = 0.807)	0.61 (0.13–2.59, *p* = 0.504)
Peripheral arterial disease	No	132 (63.5)	76 (36.5)	-	-
	Yes	8 (66.7)	4 (33.3)	0.87 (0.23–2.85, *p* = 0.823)	0.98 (0.18–4.63, *p* = 0.979)
Tuberculosis	No	138 (63.9)	78 (36.1)	-	-
	Yes	2 (50.0)	2 (50.0)	1.77 (0.21–14.98, *p* = 0.572)	0.23 (0.02–3.31, *p* = 0.273)

COPD—chronic obstructive lung disease; CKD—chronic kidney disease; T2DM—type 2 diabetes mellitus.

**Table 5:** Association between complications and COVID-19 mortality.

Dependent: Mortality		Survived	Died	OR (Univariate)	OR (Multivariate)
Tracheostomy	No	140 (77.8)	40 (22.2)	-	-
	Yes	2 (16.7)	10 (83.3)	17.50 (4.39–116.91, *p* < 0.001)	9.89 (1.78–81.48, *p* = 0.015)
Respiratory insufficiency	No	124 (80.0)	31 (20.0)	-	-
	Yes	18 (48.6)	19 (51.4)	4.22 (1.99–9.07, *p* < 0.001)	2.10 (0.73–5.69, *p* = 0.154)
Pneumothorax	No	140 (78.7)	38 (21.3)	-	-
	Yes	2 (14.3)	12 (85.7)	22.11 (5.72–146.03, *p* < 0.001)	20.91 (4.84–147.17, *p* < 0.001)
Pneumomediastinum	No	126 (75.9)	40 (24.1)	-	-
	Yes	16 (61.5)	10 (38.5)	1.97 (0.81–4.64, *p* = 0.125)	2.38 (0.87–6.47, *p* = 0.088)
Emphysema	No	107 (69.9)	46 (30.1)	-	-
	Yes	35 (89.7)	4 (10.3)	0.27 (0.08–0.71, *p* = 0.017)	0.40 (0.10–1.25, *p* = 0.141)

OR—Odds Ratio.

## Data Availability

Data available on request.
